# Ankle stiffness asymmetry is associated with balance function in individuals with chronic stroke

**DOI:** 10.1038/s41598-023-41815-w

**Published:** 2023-09-21

**Authors:** Hogene Kim, Jieun Cho, Sangwoo Cho, Joon-Ho Shin

**Affiliations:** 1 Department of Clinical Rehabilitation Research, National Rehabilitation Center, Ministry of Health & Welfare, 58 Samgaksan-ro, Gangbuk-gu, Seoul, 01022 South Korea; 2 Department of Rehabilitative and Assistive Technology, National Rehabilitation Center, Ministry of Health & Welfare, 58 Samgaksan-ro, Gangbuk-gu, Seoul, 01022 South Korea; 3 Department of Neurorehabilitation, National Rehabilitation Center, Ministry of Health & Welfare, 58 Samgaksan-ro, Gangbuk-gu, Seoul, 01022 South Korea

**Keywords:** Stroke, Biomedical engineering

## Abstract

Ankle joint is one of important contributors on balance in stroke survivors. This study aimed to investigate the relationships of ankle stiffness symmetry ratios along the talocrural and subtalar axes with clinical balance measures and weight distribution during quiet standing in ambulatory chronic post-stroke survivors. The clinical trials involved 15 ambulatory elderly with chronic post-stroke hemiparesis and 15 healthy controls. Ankle stiffness was evaluated during non-weight-bearing isokinetic passive biaxial ankle movements, and ankle stiffness symmetry ratios between paretic and non-paretic ankle stiffness (SR: Inversion/Eversion SR_IE_ & Dorsi-/Plantarflexion SR_DP_) were measured. A certified physiotherapist evaluated the Berg Balance Scale (BBS) and weight-distribution ratio (WDR) on bilateral force plates during quiet standing. Correlation coefficients, the factor analysis, and Pearson linear multiple regression were assessed with measured parameters. Correlation coefficients showed significances in-betweens; BBS and SR_DP_ (r = −0.543, p = 0.022), WDR and SR_IE_ (r = −0.667, p = 0.004), SR_IE_ and SR_DP_ (r = −0.604, p = 0.011). The exploratory factor analysis suggested four extracted factors; (1) Balance & Gait, (2) Stroke, (3) Symmetry and (4) Dimension. The first and second factors include general and pathological characteristics in stoke participants respectively. The third factor is associated with symmetrical characteristics explaining up to 99.9% of the variance. Multiple regression analysis showed ankle stiffness ratios predict BBS up to 60% of variance. The biaxial ankle stiffness ratio is a useful clinical variable that assesses balance function, in ambulatory chronic stroke survivors.

## Introduction

Balance impairments and asymmetrical movements are common outcomes of stroke hemiparesis^1^. Stroke survivors begin to regain the ability to maintain standing balance and weight-bearing symmetry in favor of the non-paretic leg; however, increased postural asymmetry and spontaneous sway, most prominently in the frontal plane, are among the most characteristic consequences of incompletely recovered hemiparesis. Asymmetric properties of the lower limb musculoskeletal characteristics in the elderly with stroke hemiparesis increase the risk of falls and fall-related injuries^2^. To prevent falls, novel simple-to-high-tech evaluations, trainings, and preventions have improved static and dynamic balance control in stroke patients using various methodologies, such as single-leg balance evaluation^3^, Tai Chi^4^, perturbation-based training^5, 6^, virtual reality^7, 8^, and affordable brain-interfaced bike^9^.

Further to the various methodological advances in hemiparetic balance, studies on asymmetric kinematics in stroke have been actively conducted on walking and standing quietly. Improvement of weight-bearing symmetry is traditionally regarded as a primary goal in stroke rehabilitation and has been associated with better motor functioning and greater independence in activities of daily living in the post-acute phase of stroke. Weight-bearing asymmetry is associated with postural instability, that is, larger center of pressure velocities; however, it does not provide evidence for a causal relationship^10^.

The symmetry ratio, that is, the ratio of paretic to non-paretic sides, has been acknowledged as a parameter to describe the complexity of asymmetry in step length and double support time in stroke survivors ^11^. Using the symmetry ratio, stance and swing time asymmetry in community ambulation among independent ambulatory stroke survivors has been reported ^12^. Stance and swing time duration asymmetry was associated with ankle muscle activation during gait ^13^ and paretic side weight-bearing during quiet standing ^14^ in stroke patients. To describe hemiparetic kinematics in stroke survivors more effectively, the symmetry ratio is known to be the adequate presentation method among the symmetry measures ^15^. Thus, the symmetry ratio is a useful tool to describe the physiological consequences, not a direct causal relationship, on hemiparetic kinematic or kinetic outcomes during gait and balance activities in stroke survivors.

Rehabilitation of the paretic ankle joint in stroke patients, one of two major strategic balance contributors, improved clinical balance measures and minor sway during gait^16, 17^. Concerning the ankle joint in healthy controls, there is experimental evidence on balance control using a simple biomechanical model with spring control at ankle plantar flexor stiffness during quiet standing^18^, ankle plantar flexor strength on dynamic balance and walking speed^19^, and ankle flexibility and plantar tactile sensation in older adults with impaired balance and functional ability^20^. Weakness of the paretic hip and knee muscles in stroke survivors mainly influences gait speed^21^, whereas studies regarding ankle rehabilitation in stroke patients have provided kinematic evidence on functional balance improvements. For example, patients with chronic stroke hemiparesis had asymmetric kinematics of paretic and non-paretic ankle joints during quiet standing in response to sway^22^. Bi-axial ankle joint stiffness-related training improves static and dynamic balance control and walking speeds^23^.

However, little is known about the relationship and internal mechanisms between asymmetric ankle properties and clinical balance capability in elderly patients with post-stroke hemiparesis. Additionally, there was no consideration of biaxial ankle movements, especially movement along the subtalar axis, that is, inversion and eversion, on balance control in ambulatory chronic stroke survivors.

This study investigates whether the asymmetric property of ankle stiffness between the paretic and non-paretic sides has a significant relationship both with clinical balance measurement in the elderly with chronic post-stroke hemiparesis and asymmetric weight distribution during bilateral standing. To express more effectively, the Berg Balance Scale and weight distribution while standing quietly as a regular daily activity were predicted using the bi-axial ankle stiffness symmetry ratios, the ratio between paretic and non-paretic ankle stiffness along bi-axial ankle movements, specifically, dorsiflexion and plantar flexion along the talocrural axis and inversion and eversion along the subtalar axis, along with participants demographic characteristics. Therefore, our hypotheses are that there are significant relationships between the biaxial ankle stiffness ratio and (1) the clinical balance measure, the Berg Balance Scale, or (2) weight-distribution asymmetry during quiet standing.

## Results

Of the 17 registered participants with chronic stoke, one did not pass the initial screening, one decided not to participate later, and one had scheduling conflicts to participate in all sessions; therefore, a total of 14 participants (five females) participated in the study. Table 1 shows demographic and clinical characteristics of the means and standard deviations of age (64.9 ± 8.9 years), body mass index (25.5 ± 2.1), time duration of stroke (119.8 ± 68.0 months), and Berg Balance Score (47.1 ± 4.7, range 39–53), together with symmetry ratios (SR_DP_: 2.05 ± 1.03, SR_IE_: 1.00 ± 0.93; weight distribution ratio (WDR): 0.97 ± 0.31), and includes the ankle stiffness symmetry ratios in age- and gender-matched healthy controls (SR_DP_: 1.03 ± 0.11, SR_IE_: 1.05 ± 0.21).

**Table 1:** Participants’ demographic and clinical characteristics.

ID	Age (years)	BMI (kg/m^2^)	Sex (M/F)	Time since stroke (mon)	Paretic side	Berg Balance Score	BBS No. 11 turning 360°	Modified Ashworth Scale	Functional ambulation category	Weight shift during quiet standing	Ankle stiffness symmetry ratio	
										ML	D/PF	I/E
S1	76	22.5	M	146.5	R	49	2	1	6	0.86	1.53	0.66
S2	68	23.1	F	109.4	R	43	2	0	6	0.74	2.08	1.54
S3	65	23.7	F	94.9	R	39	2	0	6	0.72	3.20	0.36
S4	52	26.4	M	207.4	L	53	2	0	6	0.76	1.73	0.68
S5	74	24.1	M	91.0	R	49	2	0	6	1.57	3.71	3.70
S6	78	27.7	F	105.2	L	45	2	0	6	1.13	1.05	0.06
S7	52	26.0	M	52.8	L	52	4	1	6	0.62	1.10	0.08
S8	73	24.5	M	48.1	L	41	2	0	6	1.16	2.03	0.58
S9	68	26.5	M	145.7	L	41	2	1	5	1.42	4.25	1.87
S10	74	26.3	M	42.4	R	49	2	0	6	1.10	0.82	1.03
S11	56	25.0	F	101.8	L	51	4	1	6	1.26	–	–
S12	61	24.1	M	36.6	L	52	2	0	6	1.26	1.16	1.18
S13	59	24.2	M	277.4	L	50	3	1	6	0.65	1.63	0.74
S14	54	30.4	F	142.6	L	51	4	1	6	0.70	1.99	1.01
S15	63	28.0	F	195.5	L	43	2	1	5	0.69	2.41	0.52
Mean	64.9	25.5	–	119.82	–	47.2	2.47	0.47	5.82	0.97	2.05	1.00
SD	8.98	2.12	–	68.02	–	4.71	0.83	0.52	0.35	0.31	1.03	0.93
Z	0.584	0.584	–	0.623	–	0.964	1.725 (0.005)			0.849	0.756	0.779

Healthy controls (N = 15)									Ankle stiffness symmetry ratio			
Mean	62.9	22.2	9F: 6 M	–	–	–	–	–	–	–	1.031	1.046
SD	2.5	1.15		–	–	–	–	–	–	–	0.106	0.205

*D/PF* dorsi-/plantar flexion, *I/E* inversion/eversion, *ML* mediolateral direction.

### Statistical analysis

The correlation coefficients matrix, as shown in the Table 2, there exist significant correlations between variables of symmetry; SR_DP_ vs clinical balance and gait functions (BBS: p = 0.022, FAC: p = 0.027); SR_IE_ vs standing symmetry (WDR_ML_: p = 0.005); SR_IE_ vs SR_DP_ (p = 0.011). In addition, there is a significant relationship between age and balance function (BBS: p = 0.044, BBS_11_: p = 0.006, WDR_ML_: p = 0.014).

**Table 2:** Correlation coefficients among parameters.

Age	1									
BMI	−0.337	1								
Onset	−0.325	0.145	1							
BBS	**−0.473**	0.140	0.097	1						
BBS_11_	**−0.651**	0.439	0.092	**0.463**	1					
WDR_ML_	**0.584**	−0.131	−0.428	−0.129	**−0.492**	1				
SR_DP_	0.118	−0.076	0.156	**−0.543**	−0.244	0.386	1			
SR_IE_	0.252	−0.207	−0.083	0.043	−0.232	**0.667**	**0.604**	1		
MAS	−0.351	0.300	**0.498**	0.139	**0.574**	−0.381	0.089	−0.181	1	
FAC	0.000	−0.332	−0.298	0.438	0.203	−0.135	−0.526	−0.089	**−0.471**	1
	Age	BMI	Onset	BBS	BBS_11_	WDR_ML_	SR_DP_	SR_IE_	MAS	FAC

*BBS* Berg Balance Scale, *BBS*_*11*_ BBS 11th item turning 360°, *Onset* stroke onset duration, *SR*_*DP*_ ankle stiffness symmetry ratio in dorsi-/plantar direction along talocrural axis, *SR*_*IE*_ ankle stiffness symmetry ratio in in-/eversion direction along subtalar axis, *WDR*_*ML*_ mediolateral weight distribution ratio during quiet standing, *MAS* Modified Ashworth Scale, *FAC* functional ambulation category.

Bold denotes statistical significance (p < 0.05).

The exploratory factor analysis suggested that four factors could be extracted. Bartlett’s test for dimensionality showed significance (p = 0.001) and the Communality(R^2^) showed all parameters are greater than 0.5, so all components could be used for factor analysis. Based on the Scree plot (Supplementary materials) and result of the rotated component material (Table 3), the 4-factor solution was considered further. The first factor mainly explains patients’ general balance and gait functions, explaining up to 81.4% of the variance, and the second factor is clearly related to stroke related characteristics, i.e., stroke onset duration and MAS, explaining up to 94.3%. The third factor, i.e., Asymmetry in Ankle Stiffness and Balancing Asymmetry, is related to ankle related asymmetry and measured weight distribution asymmetry, explaining up to 99.9% of the variance in the data.

**Table 3:** Exploratory factor analysis: four-factor rotated solution (varimax rotation).

Category	Item	FActor 1	FActor 2	FActor 3	FActor 4	*R*^2^
Balance & gait	BBS	0.914				0.836
	BBS_11_	0.639				0.408
	FAC	0.632				0.400
	Age	−0.544				0.296
Stroke	Onset		0.870			0.757
	MAS		0.703			0.494
Symmetry	SR_IE_			0.951		0.905
	WDR_ML_			0.727		0.529
	SR_DP_			0.718		0.516
Dimension	BMI				0.900	0.810

*BBS* Berg Balance Scale, *BBS*_*11*_ BBS 11th item turning 360°, *SR*_*DP*_ ankle stiffness symmetry ratio in dorsi-/plantar direction along talocrural axis, *SR*_*IE*_ ankle stiffness symmetry ratio in In-/Eversion direction along subtalar axis, *WDR*_*ML*_ mediolateral weight distribution ratio during quiet standing, *MAS* modified Ashworth Scale, *FAC* functional ambulation category.

**Figure 1:**
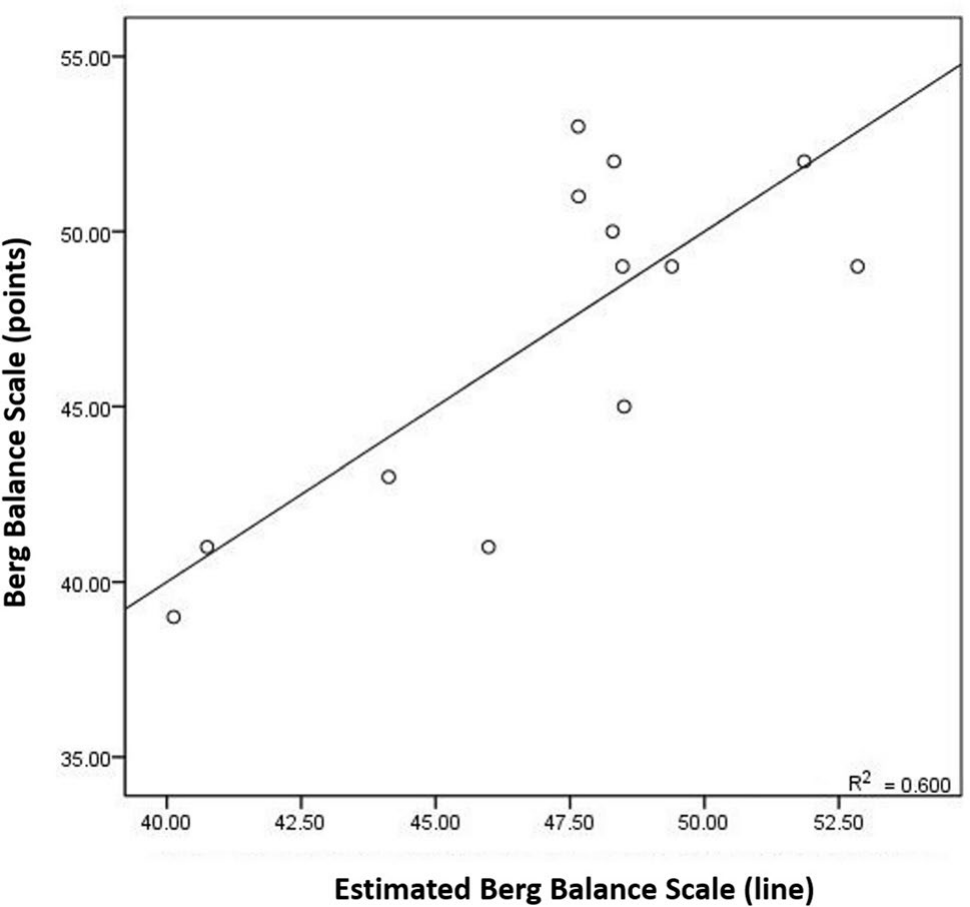
Estimated Berg Balance Scale (BBS) and weight distribution ratio during bilateral standing (WDR) using ankle stiffness symmetry ratio (SR_DP_ & SR_IE_) and demographic characteristics (age).

The regression analysis included BBS as the first outcome variable and WDR as the second outcome variable predicted by SR_DP_ and SR_IE_ and demographic characteristics including age as independent variables (Table 4). SR_DP_ was the most important predictor of BBS, explaining 29.4% of its variability alone (r = −0.543, p = 0.045), 60.0% of the variability was explained by adding SR_IE_ (Fig. 1), and explaining power increased to 78.5% by adding age (r^2^ = 0.795, p < 0.001). SR_IE_ was the most significant predictor of WDR, explaining 44.3% and 52.5% (eyes open: r = 0.666, p = 0.009; eyes closed: r = 0.725, p = 0.005) and the prevalence increased to 62.6% with age.

**Table 4:** Multiple regression analysis for Berg Balance Scale (n = 14) and weight shift during bilateral standing using ankle strength asymmetry analysis.

Variables	R	R^2^	B	SE	β	t	p	VIF
Berg Balance Scale								
Constant			71.025	5.241		13.552	< 0.001	
SR_DP_	0.295	0.236	−4.278	0.832	−9.926	−5.139	< 0.001	1.578
SR_IE_	0.512	0.423	3.803	0.948	0.741	4.010	0.002	1.662
Age	0.794	0.733	−0.292	0.079	0.550	−3.709	0.004	1.070
Weight distribution ratio (WDR) during bilateral standing								
Constant			−0.246	0.425		−0.578	0.575	
SR_IE_	−0.444	0.398	0.187	0.064	0.555	2.924	0.014	1.068
Age	0.629	0.562	0.015	0.007	0.444	2.343	0.039	1.068

*SE* standard error, *VIF* variance inflation factors.

## Discussion

The study showed that the relationship between asymmetric properties of ankle passive stiffness and clinical balance capability in the elderly with chronic post-stroke hemiparesis is significant. The age and symmetry ratio of the biaxial passive ankle joint stiffness between the paretic and non-paretic sides had a significant relationship with clinical balance measurement, that is, BBS, in ambulatory chronic stroke survivors. The custom ankle movement training device(AMT), which produces bi-directional isokinetic passive ankle movements, is useful for measuring paretic and non-paretic ankle stiffness and symmetry ratio. Furthermore, the symmetry ratio of ankle stiffness along the subtalar joint and age also have a significant relationship with the asymmetrical property during real-life balancing activities, that is, weight distribution ratio during bilateral standing with eyes either opened or closed in stroke patients, as evaluated in BBS.

The biaxial ankle stiffness symmetry ratio and age well explained the BBS and everyday balance activity, quiet standing. The value of SR_D/P_, 2.05 ± 1.03, in current participants indicates that the ankle passive stiffness in the paretic side increased about twice during over ten years in stroke hemiparesis (onset duration 119.8 ± 68.0 months) for the ankle movements along talocrural axis. According to the factor analysis, First two factors are related to patient’s general clinical evaluation on gait and balance, and stroke related clinical characteristics. The third factor would be related to symmetry ratio that related to ankle stiffness asymmetry and bilateral standing. Especially, it is apparent that the first factor, i.e., BBS & FAC, relates to ankle stiffness symmetry ratio along subtalar axis (In-/Eversion i.e., Mediolateral direction) since balance and gait capabilities have been closely related to ankle function. SR_D/P_ was negatively correlated with the BBS. Thus, as the ankle joint stiffness along the talocrural axis decreases, it is expected that it would be effective to increase the BBS, that is, improve the balancing capability. Conversely, the ankle stiffness along the subtalar axis on the paretic side would be similar to that of the non-paretic side (SR_I/E_: 1.00 ± 0.93), and the regression analysis in which ankle stiffness along the subtalar axis is positively associated with BBS. This may imply that it is effective to increase the BBS as ankle stiffness along the subtalar axis on the paretic side increases via appropriate intervention. Various reasons for increasing ankle passive stiffness in stroke survivors have been reported, such as muscle contracture or spasticity in the ankle muscles^24, 25^. However, there exists no direct study to investigate the ankle passive stiffness along subtalar axis. This CNS-mediated muscle contracture or local peripheral muscle spasticity in relatively larger ankle muscles, that is, the Gastrocnemius and Soleus, may affect passive ankle stiffness in the dorsi-/plantar direction. On the contrary, for mediolateral balance task, for example, Peroneous Longus, ankle evertor, was shown to be effective to modulate balance during gait by activating muscles along subtalar axis, which contracts through CNS-mediated dynamic signals^26^, other than the ankle passive stiffness. BBS showed a negative relationship with SR_D/P_ and a positive relationship with SR_I/E_ stiffness symmetry, indicating important information on stroke ankle rehabilitation therapies. This may imply that balance rehabilitation training, for example, fall prevention training, in ambulatory chronic stroke patients requires greater static ankle stiffness in the in-/eversion direction and more flexible static ankle stiffness in the dorsi-/plantar flexion direction. As WDR has a significant positive relationship with SR_I/E_, stiffer paretic peroneal stiffness may help stroke survivors enhance balance capability during activities of daily living. Therefore, biaxial ankle stiffness would be a useful parameter to measure, which may contribute to balancing activities in patients with chronic stroke.

Pathological asymmetry often results in fatal falls in stroke patients. Falls are a cause of critical health issues after stroke^27^. Fall incidence rates in chronic stroke survivors are higher than those in community-dwelling elderly (23–50% vs 11–30%)^28^, including higher consequences of injuries, approximately 43% fall reports, and 57% repeated falls among them^29^. Asymmetrical gait kinematics in the lower limbs of elderly individuals with chronic stroke hemiparesis have been reported in many studies^30, 31^. Impaired balancing control in the paretic lower limb contributed to gait asymmetry post stroke^14^. Kinematic and temporal asymmetries during gait are also closely related to BBS, including bilateral quiet standing^32^ and falls^33^. Therefore, the current literature and our study results would support the triangular relationship between gait asymmetry, BBS that includes bilateral quiet standing, and ankle passive stiffness asymmetric property. Therefore, in practice, paretic ankle training would be critical in stroke rehabilitation; consequently, it would be possible to prevent falls and subsequent fatal injuries in chronic stroke patients.

There is evidence that stroke hemiparesis causes altered mechanical properties in the lower limb, such as gastrocnemius spasticity and asymmetry in ankle passive stiffness, and these mechanical changes in the lower limb may further produce fatal falls. Stroke fallers exhibited higher levels of ankle muscle spasticity, which predicted falls in stroke patients using logistic regression^33^. The ankle is one of two important lower limb joints that play an important role in postural stability and kinematic balance control, the so-called ankle strategy, in addition to the hip strategy^34, 35^. While a stroke patient stands still, our results showed that the weight bearing in the paretic limb reached approximately 97% of the non-paretic limb, and its variances were 67% explained by age and mediolateral ankle stiffness symmetry ratio. In contrast, in one study, the contribution of the paretic limb to the balance task was significantly smaller than that of the paretic weight bearing^22^. Thus, proper evaluation of outcomes and therapeutic variables is significant to describe corresponding movements for balance control in stroke survivors, for example, the Berg Balance Scale for the measurement of static balance control in neurological disorders. In patients with chronic stroke, one study determined dynamic ankle mechanical impedance during the stance phase of walking and showed that ankle damping asymmetry explained 77% of the six-minute walking test distance and 65% of the ten-minute walking test speed variances, respectively. Thus, along with our current results, ankle mechanical parameters during static and dynamic movements have a significant relationship with specific clinical measures. However, this study also showed that these ankle properties had no significant relationship with common clinical lower limb measures, such as the modified Ashworth scale and Fugl-Myer assessment for lower limb^36^. Thus, specific ankle mechanical properties may have their corresponding specific clinical measures, dependent upon evaluation characteristics, for example, ankle stiffness asymmetry ratio for BBS.

Previous studies have shown that ankle sensorimotor properties successfully predict clinical balance and gait measurements in stroke survivors who are able to walk without aids. Ankle proprioception deficit was the strongest contributor to balance impairment in patients with chronic stroke^37^ [balance impaired group: BBS 40.5 (6.4)]. Ankle dorsiflexor strength is a predictor of gait velocity and temporal asymmetry during gait in stroke patients^38^. Our participant population of stroke survivors is under the acceptable to good balance categories in the BBS (39–53). Studies have suggested that the ranges of BBS in the elderly can be described as balance impairment (0–20), acceptable balance (21–40), and good balance (41–56), and is scored out of a total of 56 possible points^39, 40^. Along with the ankle proprioception deficit, the mechanical property of ankle passive stiffness symmetry was the strongest predictor of BBS. In practice, ankle sensorimotor capability significantly affects balance control and gait kinematics in patients with stroke. Thus, clinicians may be more focused on biaxial ankle strengthening and proprioceptive rehabilitation in this stroke population. Studies have been conducted on the effectiveness of ankle–foot orthoses (AFO) in stroke patients. AFO improves walking speed, cadence, step length, and stride length in patients with stroke^41^. For better applications, ankle stiffness, and its symmetry ratio would be essentially considered to be measured. As a parameter, the biaxial ankle stiffness symmetry ratio could provide clinicians with a better sense of patient treatment.

This study has limitations. First, the sample size was rather small. All stroke participants in this study had better balance capability without considerable muscle tone. Individuals who were difficult to walk independently (functional ambulatory category score of < 3) were also not included because the risk of falling during balance tasks. Therefore, the findings of this study cannot be generalized to all stroke populations. Second, careful interpretation of current results is necessary when results came from an observation study. Since there may exist other factors that affect balance, such as motor and sensory impairments, this study is limited to providing any causal relationship between the ankle stiffness asymmetry and balance function. Third, further investigation is necessary for patients with chronic stroke who had larger variations in stroke onset and functional levels with various setup, i.e., community vs clinical environment. Fourth, the reference value about the ankle stiffness symmetry ratio in healthy controls would be useful to be compared with in the future study.

## Methods

### Participants

Elderly individuals with chronic post-stroke hemiparesis were recruited from in-patient and out-patient clinics at the National Rehabilitation Center (NCR), Seoul, South Korea. The study protocol was approved by the National Rehabilitation Center Institutional Review Board, and a signed informed consent form was obtained from each participant prior to the pre-test clinical evaluation (IRB number: NRC 2015-03-020, 17/06/2015). This study was registered with the Clinical Research Information Service (KCT0002965, 29/06/2018). All study protocol and methods were performed in accordance with the relevant guidelines and regulations. The eligibility criteria included: (1) chronic post-stroke hemiparesis (stroke onset duration ≥ 1 year), (2) age between 50 and 80 years, (3) ability to walk independently on a leveled surface under supervision or using a cane (Functional Ambulation Category ≥ 3), and (4) less considerable muscle tone (Modified Ashworth Scale ≤ 2). The eligibility criteria excluded patients with (1) complications of orthopedic disorders, (2) cognitive dysfunction, and (3) mental illness.

### Test protocol and analysis

#### Clinical balance evaluations

The BBS was used to identify balance impairment. The BBS is a consistent, reliable, valid, and widely used clinical measure for balance evaluation in patients with neurological disorders^39, 42–44^. As a reliable clinical balance measure, BBS consists of 14 scored items (unable to able 0, 1, 2, 3, 4)^40^ that take about 10 to 15 min by unmediated measurement^43^. Some items required more advanced motor skills, for example, turning 360° or standing on one foot, than other items, for example, standing with feet together with eyes open. A certified physical therapist scored the BBS and supervised all the participants while performing each BBS item.

#### Stiffness measurements

The paretic and non-paretic ankle stiffness were measured using a custom biaxial ankle movement training device (AMT). The AMT was developed to reliably and repeatedly measure ankle joint stiffness during non-weight-bearing biaxial isokinetic passive ankle movement^45^. Participants were comfortably seated on a height-adjustable chair with 90° knee flexed and the target paretic or non-paretic foot fastened on the foot cradle in the AMT. In this posture, participants were able to avoid awkward training posture, e.g., supine or prone position, and to reduce weight bearing on ankle joint while training in AMT. When the AMT starts moving along the ankle and subtalar axes (talocrural axis for dorsiflexion and plantarflexion, subtalar axis for inversion/eversion), it moves at slow speed (average 2.14°/s, sd:0.43°/s), not to cause joint spasticity, and the custom foot force plate in the foot cradle measures the ground reaction force and torque along the ankle and subtalar axes sampled at 100 Hz. The foot force plate was implemented using two aluminum plates and four bar-type load cells, which were diagonally placed at four corners in between them. During testing, a motorized seesaw-type foot cradle controlled by an independently moving 42°-tilted motor attached to the foot cradle provided ankle dorsi-/plantarflexion movements along the ankle axis, inversion/eversion movements along the subtalar axis, and their combinations (Supplementary video).

While the AMT moves the full range along a given joint, the force place on the cradle measures torque. The slope of torque-joint angle was defined as the passive joint stiffness. We first assume that ankle passive torque during ± 20° in dorsi-/pantarflexion direction (along talocrural axis) and ± 12° in in-/eversion direction (along talocalcaneal or subtalar axis) have a linear profile. The average slope is the ankle passive stiffness in this study. Detail definitions and calculation methods of the ankle passive stiffness and its symmetry ratio were described in the supplementary material.

#### Symmetry ratios

The symmetry ratio (SR) has successfully been an effective parameter to compare affected and non-affected individuals in many studies on the kinematics and kinetics of balance, posture, and gait in people with neuromusculoskeletal diseases^15, 46–50^. The SR between paretic and nonparetic ankle stiffness in stroke patients was defined as follows:

1 $$Symmetry\, Ratio \left(SR\right)=\frac{Paretic\, Side}{NonParetic\, Side}$$

The Eq. (1) of ankle passive stiffness was measured in the AMT during bi-directional isokinetic passive movements (SR in ankle passive stiffness; (1) SR_DP_ of ankle passive stiffness in dorsi-/plantarflexion along the talocrural axis and (2) SR_IE_ of ankle passive stiffness in inversion/eversion along the subtalar axis). Passive stiffness was measured during bi-axial isokinetic ankle movements between 20°and the participant’s own ankle range of motion in both paretic and non-paretic sides^45, 51, 52^ and subsequently, the SR was calculated.

When BBS was evaluated for the items of standing still with eyes open and closed (BBS No. 6 and 7) in stroke participants, the weight distribution was recorded for 30 s three times while standing comfortably with each foot on two bilateral force plates, sampled at 1000 Hz (AMTI, Watertown, MA, USA). During these evaluations, the mediolateral weight distribution ratio (WDR), Eq. (2), was evaluated by the ratio of weights on the paretic and non-paretic sides during comfortable bilateral standing on two force plates with eyes open and closed, respectively.

2 $$WDR=\frac{{Weight(W)}_{ Paretic\, side}}{{Weight(W)}_{ NonParetic\, side}}$$

### Statistical analysis

SPSS software (version 21.0) for Windows (SPSS, Inc., Chicago, Illinois, USA) was used for statistical analysis. Descriptive statistics were used for all equipment and demographic characteristics. Pearson linear correlation coefficients was constructed between parameters to evaluate the relationships among parameters, i.e., the asymmetrical ankle properties, BBS, WDR, MAS etc. The factor analysis was conducted to explore the underlying structure of the relationship with ankle asymmetry and parameters. A multiple regression analysis was conducted to determine the independent predictors of both the BBS and WDR. The significance level for all statistical tests was set at P < 0.05.

### Supplementary Information

Supplementary Video 1.

Supplementary Information.

## Data Availability

The datasets generated during and/or analysed during the current study are available from the corresponding author on reasonable request.
