# Supplementary material for: Ankle stiffness asymmetry is associated with balance function in individuals with chronic stroke
Source: Sci Rep. 2023 Sep 21;13:15721. doi: 10.1038/s41598-023-41815-w (PMC10514256; doi:10.1038/s41598-023-41815-w)
Supplement: Supplementary file 2 — Supplementary Information. [file 41598_2023_41815_MOESM2_ESM.docx]

**Definition of Ankle Stiffness Symmetry Ratio**

ii) At given ankle angle, the torque was calculated as shown below

1. **Definition of paretic and non-paretic ankle stiffness**

The average stiffness is defined as the slope of torques at given ankle angles; θ_1_ and θ_2_ along a given axis

$$F_{\theta i,F}$$

$$T_{\theta i}$$

$$l_{0}$$

$$l_{0}$$

$$F_{\theta i,H}$$

$$T_{\theta i}=\left( F_{\theta i,F}-F_{\theta i1,H} \right)l_{0}$$

θ

**Torque**

θ_1_

θ_2_

T_θ1_

T_θ2_

Paretic Full Range of Motion (ROM_p_)

Paretic stiffness curve $k_{p}$

Non paretic stiffness curve $k_{np}$

Non paretic Full Range of Motion (ROM_np_)

$$k_{p}$$

$$k_{np}$$

where $\theta_{1}-\theta_{2}=\pm20^{\circ} along talocrural axis$

$=\pm$12$^{\circ} along subtalar axis$

iv) **Definition of Ankle Stiffness Symmetry Ratio**
 between paretic and non-paretic sides

${Symmetry Ratio}_{ankle Stiffness}=\frac{k_{P}}{k_{nP}}$

$=\frac{\left[ \left( F_{\theta1,F}-F_{\theta1,H} \right)l_{0}-\left( F_{\theta2,F}-F_{\theta2,H} \right)l_{0} \right]_{P}}{\left[ \left( F_{\theta1,F}-F_{\theta1,H} \right)l_{0}-\left( F_{\theta2,F}-F_{\theta2,H} \right)l_{0} \right]_{nP}}$

$=\frac{{\Delta F}_{P}}{{\Delta F}_{nP}}$

$$k_{ankle}=\frac{\Delta T}{\Delta\theta}=\frac{T_{\theta1}-T_{\theta2}}{\theta_{1}-\theta_{2}}=\frac{\left( F_{\theta1,F}-F_{\theta1,H} \right)l_{0}-\left( F_{\theta2,F}-F_{\theta2,H} \right)l_{0}}{\theta_{1}-\theta_{2}}$$

iii) Calculation of mean ankle stiffness (The torque slope)
